# The Zymark BenchMate™. A compact, fully-automated solution-phase reaction work-up facility for multiple parallel synthesis

**DOI:** 10.1155/S1463924600000328

**Published:** 2000

**Authors:** Gordon A. Hamlin

**Affiliations:** AstraZeneca Alderley Park Cheshire Macclesfield SK10 4TG UK

## Abstract

The rapid growth of multiple parallel synthesis in our laboratories has created a demand for a robust, easily accessed automated system for solution-phase reaction work-up, since the manual work-up of large numbers of small-scale reactions is both time-consuming and tedious, and is a rate limiting step in the generation of large numbers of compounds for test. Work-up in chemical organic synthesis consists of a series of post-reaction operations designed using differential chemical properties to remove excess reagent or starting material, reagent products and, where possible reaction by-products. Careful consideration of post-reaction operations as a clean-up step can obviate the requirement for purification. Generally, work-up can be resolved into four operations: filtration, solvent addition (dilution, trituration), washing and separation (partition) and it is the selection and ordering of these four basic operations that constitutes a chemical work-up. Following the proven success of centralized Zymate robotic systems in the compilation, execution and work-up of complex reaction sequences, a centralized chemical work-up service has been in operation for over 12 months. It now seemed prudent that the needs of multiple parallel synthesis would be better served by the development of a compact, automated system, capable of operating in a standard chemistry laboratory fume-hood. A Zymark BenchMate platform has been configured to perform the four basic operations of chemical solution work-up. A custom-built filtration station, incorporating an integrated tipping facility for the sample tube has also been developed. Compilation of each work-up is through a set of Visual Basic procedure screens, each dedicated to a particular work-up scenario. Methods are compiled at the chemist's own PC and transferred to the BenchMate via a diskette.


Journal of Automated Methods & Management in Chemistry, Vol. 22, No. 6 (November–December 2000) pp. 181–186

### The Zymark BenchMate™. A compact, fully-automated solution-phase reaction work-up facility for multiple parallel synthesis

**Gordon A. Hamlin**
*AstraZeneca, Alderley Park, Macclesfield, Cheshire, SK10 4TG, UK*

*The rapid growth of multiple parallel synthesis in our laboratories has created a demand for a robust, easily accessed automated system for solution-phase reaction work-up, since the manual work-up of large numbers of small-scale reactions is both time-consuming and tedious, and is a rate limiting step in the generation of large numbers of compounds for test. Work-up in chemical organic synthesis consists of a series of post-reaction operations designed using di erential chemical properties to remove excess reagent or starting material, reagent products and, where possible reaction by-products. Careful consideration of post-reaction operations as a clean-up step can obviate the requirement for purification. Generally, work-up can be resolved into four operations: filtration, solvent addition (dilution, trituration), washing and separation (partition) and it is the selection and ordering of these four basic operations that constitutes a chemical work-up. Following the proven success of centralized Zymate robotic systems in the compilation, execution and work-up of complex reaction sequences, a centralized chemical work-up service has been in operation for over 12 months. It now seemed prudent that the needs of multiple parallel synthesis would be better served by the development of a compact, automated system, capable of operating in a standard chemistry laboratory fume-hood. A Zymark BenchMate platform has been configured to perform the four basic operations of chemical solution work-up. A custom-built filtration station, incorporating an integrated tipping facility for the sample tube has also been developed. Compilation of each work-up is through a set of Visual Basic procedure screens, each dedicated to a particular work-up scenario. Methods are compiled at the chemist's own PC and transferred to the BenchMate via a diskette.*

### Introduction

Chemical synthesis can be divided into three distinct phases; reaction, work-up and purification. All three areas have been the subject of automated methodology with robotic reaction compilation and execution, along with post reaction separation techniques using Zymark systems, becoming widely used in the batch-wise generation of large numbers of compounds for, mainly pharmaceutical, screening.

Within the past three years there has been a dramatic increase in the use of multiple parallel synthesis (MPS) where the individual chemist carries out a parallel series of, typically ten to twenty, reactions using a mixture of apparatus to effect stirring with heating or cooling, manual filtration with washing and solid phase extraction (SPE). Whilst there is a range of heater/stirrers, vacuum filtration tanks and multiple, pumped BondElut (SPE) systems, some with fraction selection, the role of work-up, i.e. filtration, dilution and liquid/liquid extraction has been largely left in the hands of the chemist. Liquid handling XYZ robots have been evaluated for this role but their main drawback is their inability to collect solids by filtration or homogenize immiscible liquids and effect timely extraction.

Following the proven success of centralized Zymate robotic systems in the compilation, execution and work-up of complex reaction sequences, a centralized chemical work-up service has been in operation for over 18 months [1]. It now seemed prudent that the needs of MPS would be better served by the development of a compact, automated system, capable of operating in a standard chemistry laboratory fume-hood. Adapting a cylindrical XP robot to operate in the confined space was disqualified by the inherent action of the arm whereby operation at minimum reach necessitates a similar volume of 'elbow' room behind. The Zymark BenchMate platform, currently designed for quality assurance liquid-handling, operates within a confined volume (less than $1 m^3$) using a hand cantilevered under the arm. The penalty for this is the loss of circular wrist action, important for 'pouring' during, for example, filtration.

Thus, a Zymark BenchMate platform was configured to perform the operations of chemical solution work-up. A custom-built filtration station, incorporating an integrated tipping facility for the sample tube was included (figure 1).

Compilation of each work-up is through a set of Visual Basic procedure screens, each dedicated to a particular work-up scenario. Methods are compiled at the chemist's own PC and transferred to the BenchMate via a diskette.

### Work-up

Work-up in chemical organic synthesis consists of a series of post-reaction operations designed using differential chemical properties to remove excess starting material, reagents or reagent products. Purification is generally the separation of the product of the reaction from reaction by-products. Careful consideration of post-reaction operations as a clean-up step can sometimes obviate the requirement for purification. This has been an attractive feature of some MPS at Alderley Park, which we have attempted to address with automated procedures. Generally, work-up can be resolved into four basic opera-

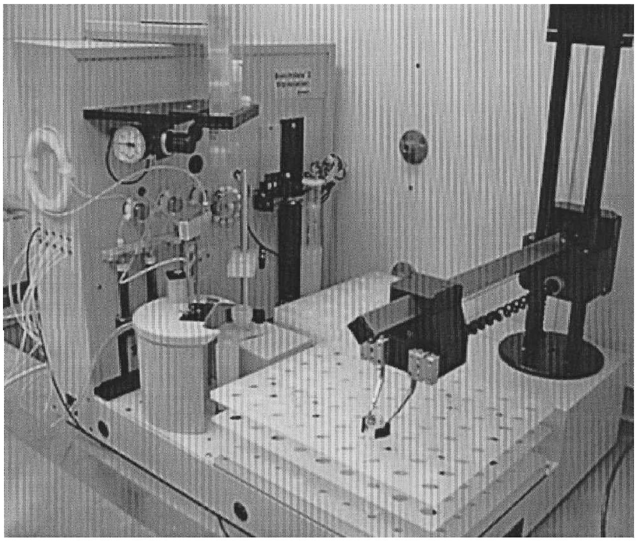

*Figure 1. The custom BenchMate II workstation.*

tions: filtration, solvent addition (dilution, trituration), washing and separation (partition) and it is the ordering and selection of these four basic operations that constitutes a work-up procedure.

- *Filtration.* This involves the separation (collection) of solid material by passing a slurry of solid and liquid material through a suitable membrane. The filtered residue may be washed free of the liquid by washing with a suitable solvent. The clarified liquid and washes may be combined to form the filtrate.
- *Solvent addition.* This is the addition of solvent for the purpose of dilution, dissolution or partial dissolution followed by filtration.
- *Washing.* This invokes the transfer of solutes between immiscible layers by the agency of relative solubility (partition) or by the use of managed chemical conditions (extraction). During this operation it is necessary to effect efficient mixing in order to maximize inter-phase surface area contact.
- *Separation of layers.* This is the physical removal of either the upper or lower layer from a two-phase system. These layers may be the product of a chemical reaction mixture or have resulted from washing one solution with another (see Washing).
- *Partition.* This is a combination of *washing* and the *separation of layers.*
- *Trituration.* This involves the *addition of solvent* to a solid or a gum in order to separate the desired product from impurities by partial dissolution followed by filtration.

Zymark XP systems in the centralized Robotics facility at Zeneca Pharmaceuticals, Alderley Park [1–3] have demonstrated their ability to efficiently execute these operations.

### Work-up Scenarios

The BenchMate is configured to execute the following protocols.

- *Filter solids and wash the filter.* The slurry to be filtered is fluidized by vortex mixing before being tipped into a 20 ml Whatman AutoCup filter. A Nylon membrane is employed for aqueous slurries, PTFE for organic mixtures. Air pressure (5–10 psi) is applied for a selected time and the filtrate collected in a collection tube. The sample tube may now be washed up to three times by the addition of solvent and vortex mixing. All washes are passed through the filter into a combined filtrate.
- *Filter solids and wash (partition) the filtrate.* The procedure above is executed to remove solid material before the combined filtrate is subjected to washing (vortex mixing and settling) and separation (partition) by up to three aqueous solutions. The washed organic layer may now be dried by passing through a filter which has been pre-loaded with granular magnesium sulphate with the option to wash the filter.
- *Wash (partition) an organic solution.* This scenario allows the option of first adding an organic solvent with a view to either diluting an existing solution or changing the aqueous miscibility characteristic of a solution. As above, it then allows the organic layer to be washed up to three times with a variety of aqueous solutions before drying.
- *Extract an aqueous solution.* The pH of an aqueous solution may be altered by the addition of a measured amount of solution of acid or base. The resulting mixture may then be extracted up to twice with an organic solvent (vortex mixing, settling of layers and separation of layers). The combined organic extract may now be back-washed with an aqueous solution before drying as before.
- *Dissolve or triturate a solid/gum.* A solid or a gum (typically the product of an evaporated reaction mixture) may be treated with either an aqueous solution or an organic solvent and vortex mixed for a selected time to effect either complete or partial dissolution. The resulting mixture may then be treated in a variety of ways.

  (1) A slurry may be filtered and the solid material washed and collected.
  (2) A slurry may be filtered, the filter washed and the organic filtrate washed with up to three aqueous solutions. The washed organic layer may then be dried (as before).
  (3) A solution may be washed with up to three aqueous solutions and the washed organic layer dried (as before).

### Hardware

See figure 2.

### *Racking and tubes*

The BenchMate is configured to operate with sample populations of up to 72 (20 × 125 mm) tubes (single tube operations; dissolve/dilute etc.), 32 tubes (two tube procedures; filtration/partition etc.), 24 tubes (three tube procedures; filtration with partition, partition with dry-

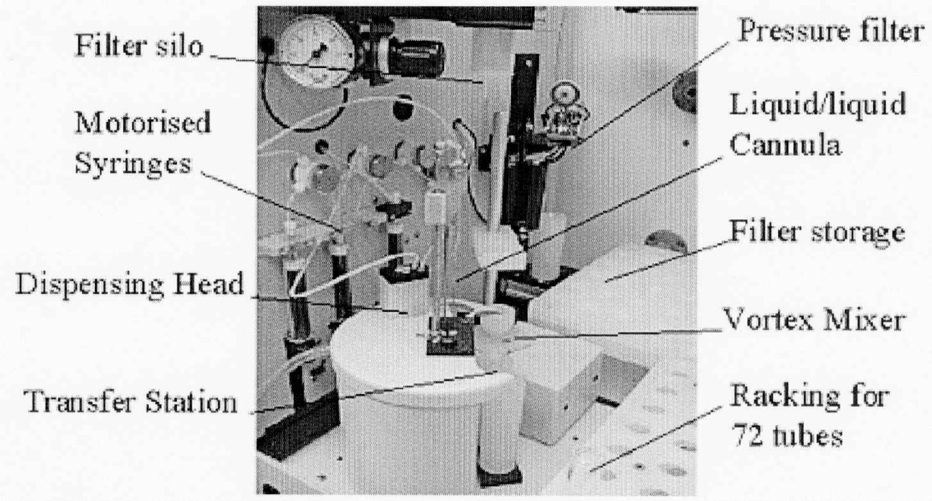

*Figure 2. Layout of the hardware used.*

ing etc.) and 16 tubes (four tube methods; filtration, partition and drying). The single 72-position rack is delineated into up to three racks: sample rack, transfer rack and collection rack, according to the method. The final product solution is always returned to the sample rack.

*Dispensing*

Currently, it will dispense up to six aqueous solutions via a 12-port valve and one organic solvent. Dispensing is driven by a pair of motorized syringes. When a new solution is dispensed, the line from the 12-port valve to the cannula is purged automatically. Liquids are delivered into the tubes when the dispensing head rotates to a position over the tube in the transfer station.

*Liquid storage*

Liquids (solutions, solvents and cannula wash medium) are delivered from capped bottles through dedicated lines.

*Mixing*

Agitation is by vortex mixing to homogenize layers during dilution, dissolution, partition or to fluidize magma prior to filtration. The length of time of mixing can be set by the operator.

*Filtration*

Filtration is by air pressure (5 psi) through Whatman 20 ml filter cups (PTFE and Nylon). The sample tube is held in the tipping-station by vacuum applied to a cup into which the base of the tube sits. After fluidization the mixture is poured into the filter using a specially designed tipping mechanism (figure 3). The rate at which the sample tube tips is controlled by hand. The tube may be washed into the filter up to three times. Filters are delivered from vertical silos, with separate columns for product and drying filters and finally stored on a flat rack (with individual drip vials) after filtration. Filtration time may be reset by the operator.

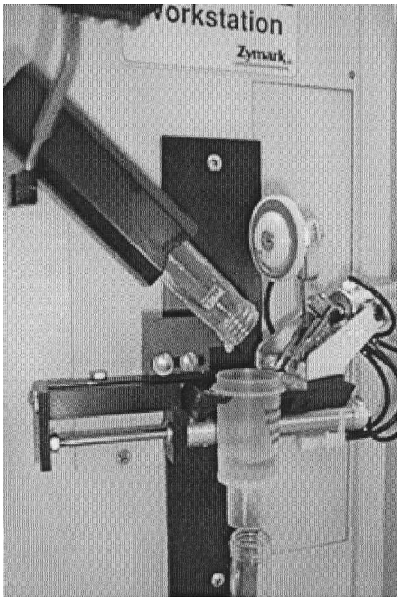

*Figure 3. Mixture being poured into the filter.*

*Drying*

Drying organic solutions is by filtration (as above) through a drying medium ($MgSO_4$, Hydromatrix Earth etc.) as the final step of a procedure.

*Separation of layers*

Separation of layers during partition is through a vertical cannula. Aspiration is by a dedicated motorized syringe and the liquid removed is stored in a 10 ml loop before dispensing into a second tube. Where possible, the lower layer is aspirated. The volume of the aspirated layer may

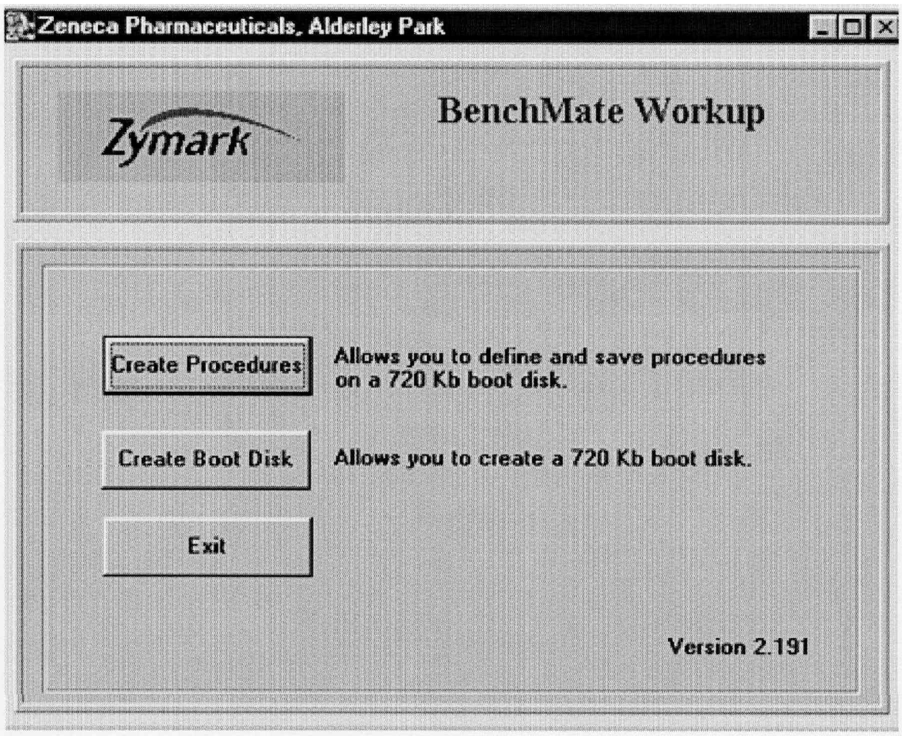

*Figure 4. Entry screen.*

be set by the operator (to accommodate volume changes during partition) or left at the default values of the relevant wash volumes added during the procedure.

### Software

*Operator Interface*

**Work-up method parameters** are entered off-line at the individual chemist's own PC through a series of Visual Basic menus. The method is then saved to a 3.5″ DS/DD 720 diskette which is transferred to the BenchMate in order to run the method.

**Methods are compiled** by selecting one of a series of Method Options (see below) which directs the operator to a particular procedure screen wherein a specific work-up is tailored from a range of operations, solvent/solution choices and volumes.

*The entry screen (figure 4) allows the operator to:*

- exit the application;
- create a boot disk containing all the operating system files necessary to run the BenchMate as well as the EasyLab dictionary containing the programs required to run the workup methods;
- create a new procedure by saving parameters to a boot disk. This option takes the operator to a series of procedure screens (see below) and allows the construction of a specific method which is then saved to the boot disk.

*Procedure screens*

- The relevant procedure screen is selected by choosing from a series of Method Options, viz:

  (i) filter and wash filter;

  (ii) filter and wash filtrate;

  (iii) wash an organic solution;

  (iv) wash an aqueous solution;

  (v) separate layers and wash organic.

  (vi) dilute, dissolve, triturate.

- In all procedure screens, sample information, i.e. total number of samples, initial volume in the sample tubes and starting tube number is set in the first part of the Visual Basic screen.
- The remainder of the screen is divided into areas dealing with specific operations, e.g. (see figure 5) optional dilution with organic solvent, number and nature of aqueous washes and optional drying by filtration through a drying agent.
- All screens display a Method Description which defines the core procedure and the range of options. It also gives instruction on the number of tubes required per sample and how these tubes should be arranged (see racking and tubes).

### Operation

*Software*

After inserting the diskette into the BenchMate disk drive, pressing the LOAD button loads the diskette's software into the CPU and automatically starts a procedure. Pressing the PAUSE button stops the workstation after it completes the step it is performing. The CONT button continues operation after using the PAUSE key or after responding to the ERROR light. The ERROR light is activated when a condition that

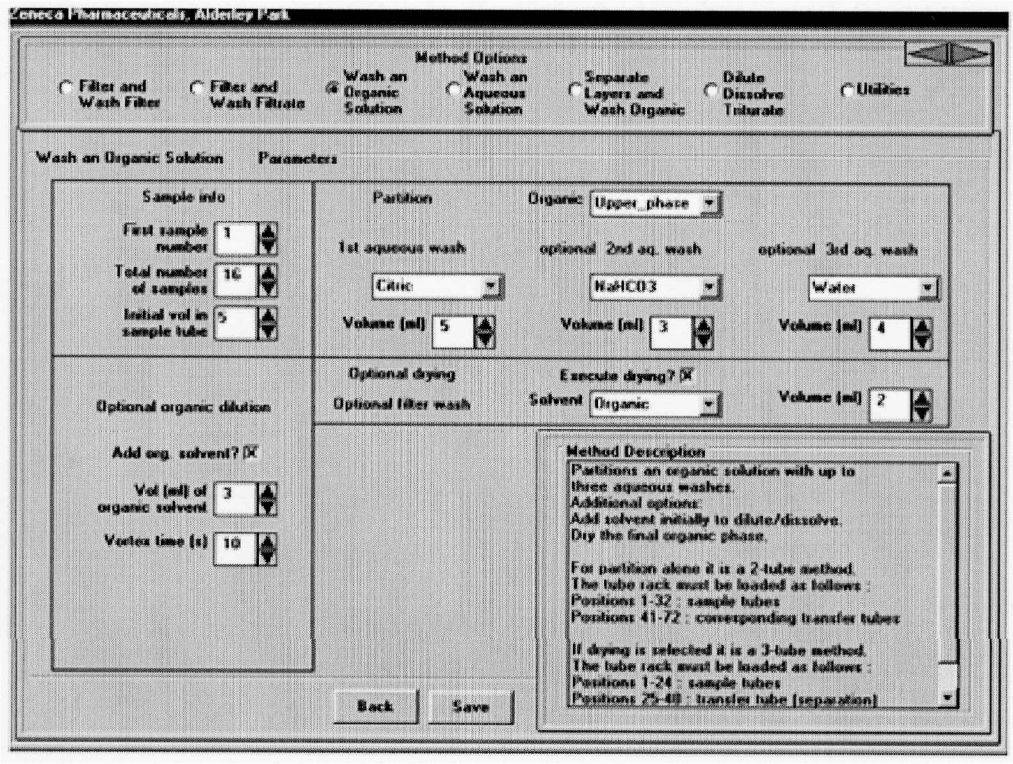

*Figure 5. Procedure screen.*

affects successful operation is detected. A descriptive error message is also written to the diskette.

*Hardware*

The operator must ensure that sample tubes, transfer tubes and collection tubes are in the correct positions, that the relevant reservoirs are sufficiently full and that the filter silo is charged and the filter collection rack is empty.

### Chemistry

1. *Mitsonobu cyclization (Bu3P/ADDP)*

   5 samples: dissolve evaporated reaction mixture in EtOAc (5 ml),
   wash with water (4 × 5 ml),
   wash with aqueous citric acid (1 × 5 ml),
   wash with aqueous, sodium bicarbonate (1 × 5 ml),
   dry the organic layer by filtration through $MgSO_4$.

2. *Coupling reaction (EDC/HOBT)*

   12 samples: filter 2-phase mixture,
   separate layers,
   wash organic layer with citric acid (1 × 5 ml),
   wash organic layer with water (1 × 5 ml).

3. *Coupling reaction (EDC/HOBT)*

   18 samples: dissolve evaporated reaction mixture in EtOAc (6 ml),
   wash organic layer with citric acid (1 × 5 ml),
   wash organic layer with water (1 × 5 ml),
   dry by filtration through $MgSO_4$.

4. *Displacement of a chloro-heterocycle with arylalkylamines*

   12 samples: filter solids and wash filter with water.

5. *Displacement of a chloro-heterocycle with phenols*

   10 samples: dissolve evaporated reaction mixture in EtOAc (8 ml),
   wash organic layer with water (4 ml)
   dry by filtration through $MgSO_4$.

6. *Coupling reaction (EDC/DMAP)*

   5 samples: dissolve evaporated reaction mixture in $CH_2Cl_2$ (8 ml),
   wash organic layer with 5% potassium carbonate (2 × 4 ml),
   dry by filtration through $Na_2SO_4$.

7. *Coupling reaction (HATU coupling reagent).*

   12 Samples: dissolve evaporated reaction mixture in EtOAc (8 ml), plan to wash with water run intercepted when solids precipitated after initial addition of EtOAc,
solids were collected by filtration.

8. *Coupling reaction* (*EDC*)

8 Samples: triturate the evaporated reaction mixture with water (6 ml, $2 \times 3$ ml),
solids collected by filtration.

### Summary and conclusions

A custom BenchMate configured for the operations of solution-phase reaction work-up has been described. Working in populations of up to 72 ($20 \times 125$ mm) tubes the BenchMate will deliver precise volumes of solutions and solvents, agitate to effect maximal partition between immiscible solvents and, after the layers have settled, accurately aspirate either the upper or lower layer. The aspirated layer is then dispensed into a collection tube. Organic layers may be dried by passage through a drying agent. Aqueous or organic slurries are filtered by tipping the contents without spillage into a Whatman AutoCup before filtering with positive air pressure. Combinations of the above operations constitute a work-up.

The use of the custom BenchMate by chemists engaged in multiple parallel synthesis will bring relief from the tedious, manual routines of work-up. Consideration is currently being given to the development of the custom BenchMate into the fields of solution-phase reaction compilation and incubation, solid-phase reaction execution, and solid-phase work-up using either scavenger resins or solid-phase extraction (silica and ion exchange).

### Acknowledgements

The author acknowledges the invaluable contribution of David Rudge and Mandy Crowther (AstraZeneca) to his understanding of chemical synthesis robotics and Easylab programming, and Ian Nash and Kurt Pike (AstraZeneca) for their support and suggested improvements during the realization of this project. Thanks also to Terry Roche and Elise Corrigan (Zymark UK) for converting this concept into reality.

### Trademarks

Zymark, Zymate, System V, BenchMate and EASYLAB are registered trademarks of Zymark Corporation.

### References

1. Hamlin, G. A., 1998, A versatile, fully automated, open access work-up station designed to serve the needs of the rapidly expanding field of multiple parallel synthesis. *Proceedings of the International Symposium on Laboratory Automation and Robotics*.
2. Main, B. G. and Rudge, D. A., 1994, Automated chemical synthesis. *Proceedings of the International Symposium on Laboratory Automation and Robotics*.
3. Rudge, D. A. and Crowther, M. L., 1997, Development of workstations for filtration and purification of samples prepared by automated syntheses. *Proceedings of the International Symposium on Laboratory Automation and Robotics*.

